# Individual and community-level factors affecting diabetes screening uptake among adults in 14 low- and middle-income countries: a multilevel mixed-effects analysis of recent demographic and health surveys

**DOI:** 10.1186/s12889-026-28221-3

**Published:** 2026-07-10

**Authors:** Kegnie Shitu, Kate McBride, David Simmons, Freya MacMillan

**Affiliations:** 1 School of Health Sciences, Western Sydney University, Campbelltown, NSW 2560 Australia; 2 College of Medicine and Health Sciences, University of Gondar, Gondar, Ethiopia; 3 School of Medicine, Western Sydney University, Campbelltown, NSW 2560 Australia; 4 Translational Health Research Institute, Western Sydney University, Campbelltown, NSW 2560 Australia; 5 Division of Research and Innovation, Western Sydney University, Penrith, NSW 2751 Australia

**Keywords:** Diabetes screening, Health behaviour, Low‑ and middle‑income countries, Undiagnosed diabetes

## Abstract

**Background:**

Diabetes screening is vital for the early detection and management of the condition, aiming to improve prognosis and prevent disease progression and complications. However, evidence on population‑level uptake of diabetes screening and its determinants in low‑ and middle‑income countries (LMICs) remains limited. Therefore, this study assessed uptake of diabetes screening and the associated individual‑ and community‑level factors among adults in LMICs.

**Methods:**

We analysed recent Demographic and Health Surveys from 14 LMICs across Africa, South-East Asia, and the Americas. Weighted prevalence estimates of individuals who have ever undergone diabetes screening were calculated for each included country, by participant characteristics. Multilevel logistic regression models were fitted for each country to examine individual- and community-level determinants of diabetes screening uptake. Community variances, intraclass correlation coefficients, and proportional change in variance were used to measure community-level variability in diabetes screening uptake. Deviance, Akaike information criterion, and Bayesian Information criterion were used to select the best-fitting model for each survey. Adjusted odds ratios were used to estimate the association between covariates and diabetes screening uptake.

**Results:**

The prevalence of diabetes screening uptake varied widely across countries, ranging from 5.9% in Timor-Leste to 39.8% in the Dominican Republic. In most countries, women had significantly higher screening uptake than men. Older age, higher educational attainment, marital status, health insurance coverage, media exposure, and prior blood pressure measurement were consistently associated with increased likelihood of diabetes screening uptake across multiple surveys. At the community level, higher community media exposure and, in some contexts, higher community educational attainment were associated with greater odds of screening uptake. Substantial between‑community heterogeneity was observed, with intraclass correlation coefficients ranging from 6.8% to 40%.

**Conclusion:**

Diabetes screening uptake remains suboptimal across many LMICs, with pronounced disparities by sex, socioeconomic status, and community characteristics. Strengthening health system performance, expanding health insurance coverage, increasing community‑level awareness, and better integrating diabetes screening into routine primary care may improve early detection. Addressing both individual‑ and structural‑level determinants is essential to enhancing screening uptake, reducing the burden of undiagnosed diabetes, and advancing global targets to mitigate diabetes‑related complications.

**Supplementary Information:**

The online version contains supplementary material available at https://doi.org/10.1186/s12889-026-28221-3.

## Background

Diabetes is one of the major non-communicable diseases affecting 589 million adults worldwide in 2024, estimated to rise to 853 million by 2050. In addition to this, 634.8 million adults are at high risk of developing diabetes (T2D), 70–90% of whom are likely to progress to T2D during their lifetime if preventative measures are not successfully undertaken [1]. Globally, diabetes caused one death every nine seconds and led to significant health expenditure (one trillion dollars) in 2024 [2]. Even though diabetes affects people living in all parts of the world, some regions are disproportionately affected by the condition. According to the 2025 International Diabetes Federation report, in 2024, most adults with diabetes lived in LMICs, totalling 474.7 million, compared with 114.1 million in high-income countries. This figure is expected to increase fivefold in LMICs compared to high-income countries by 2050, with projected rises of 52.9% and 10.8%, respectively. Despite this health disparity, diabetes treatment coverage is the lowest in LMICs, resulting in disproportionately higher rates of diabetes complications such as kidney failure, blindness, cardiovascular disease, and limb amputations [3].

Despite global efforts to prevent and manage diabetes and its complications, a substantial proportion of individuals worldwide continue to live with undiagnosed diabetes, placing them at increased risk of severe complications and premature mortality [4]. Globally, an estimated 42.8% of adults with diabetes were undiagnosed in 2024 [2]. Approximately 87% of adults worldwide with undiagnosed diabetes are estimated to live in LMICs, who could experience an even greater risk of complications and premature death due to regionally constrained health systems [2]. For example, a study across multiple countries found the highest prevalence of undiagnosed diabetes among adults in Africa (53.6%), the Western Pacific (52.8%), and South-East Asia (51.3%) in 2021 [5]. These figures have risen considerably, especially in the African region and low-income countries overall, where, in 2024, the prevalence of undiagnosed diabetes among adults was estimated to be 72.6% and 58%, respectively [2]. This disparity could be due to differences in health system performance, socioeconomic status, health literacy, the diabetes screening approaches, or individuals’ screening behaviour [6]. Moreover, recent evidence indicates that obesity and other T2D risk factors have increased substantially over recent decades in most LMICs, although the magnitude of increase varies across settings; for instance, age-standardised obesity prevalence increased almost nine-fold in South-East Asia from 0.9% in 1980 to 7.9% in 2019, while obesity more than doubled among adults in Southern African countries between 1990 and 2019 [7, 8], which is likely to lead to even more undiagnosed diabetes cases, highlighting the importance of diabetes screening [9].

Diabetes screening is a key factor in early diagnosis and the prevention of complications. Effective screening enables the timely initiation of treatment, can reduce the progression of the condition among high-risk people through access to treatment, and can help people with diabetes avoid or delay complications and premature mortality due to late diagnosis, while preserving quality of life [10]. Early detection and management of diabetes can also mitigate the substantial healthcare costs associated with diabetes and its complications [2]. Nonetheless, diabetes screening in LMICs remains limited, with substantial losses occurring at the testing stage and a large proportion of cases remaining undiagnosed [11]. Screening strategies in these settings are often targeted or opportunistic, which may limit the reach of screening programmes, unlike population-based screening that can reach a broader segment of the population and support earlier detection of diabetes. In addition, the limited availability of convenient diabetes screening modalities affects screening uptake [11, 12]. Compared with fasting plasma glucose (the commonest diagnostic test available in most LMICs), HbA1c is more convenient because it does not require fasting and can often be completed in a single visit [13]. However, access to HbA1c testing remains limited in many LMIC settings, which could eventually affect diabetes screening uptake [14].

Despite limited comprehensive data on community-based diabetes screening uptake in LMICs, available research suggests that early detection of diabetes remains limited. A multi-national study assessing diagnostic testing for cardiovascular risk factors found that health systems across 57 LMICs were underperforming in adhering to the World Health Organisation (WHO) Package of Essential Noncommunicable Disease Interventions (PEN) standards for primary care. This study found that only 45% of individuals who met the WHO PEN criteria for diabetes screening had been appropriately tested, suggesting that a substantial proportion of individuals with diabetes remain undiagnosed [15]. Another study conducted in South Africa found that among adults who had never had their blood sugar checked, 10% of males and 6% of females had HbA1c results indicating diabetes, and that 67% of both genders were at high risk of developing diabetes (based on the presence of pre-diabetes with an HbA1c of 5.7%-6.4%), highlighting the importance of diabetes screening [16]. Along with enhancing diabetes-related services in primary healthcare systems, encouraging individuals’ uptake of diabetes screening is a key strategy for prevention and early detection, especially in resource-limited settings with low health literacy.

In order to reduce the health and economic impact of undiagnosed diabetes, the WHO, through the Global Diabetes Compact, has established global coverage targets, including diagnosing 80% of people with diabetes by 2030 [17]. This target is likely to be hard to achieve unless health systems’ performance is improved, scalable primary care-based diabetes screening is in place, and the public’s health awareness and screening uptake are enhanced, especially in resource‑limited settings where diagnostic gaps remain large [5].

Given the paucity of data from LMICs, understanding the extent of diabetes screening uptake and the individual‑ and contextual‑level determinants that influence it is essential for informing effective public health strategies to increase screening uptake and reduce the burden of undiagnosed diabetes and its consequences. Accordingly, this study examined individual‑ and community factors associated with diabetes screening uptake among adults in 14 LMICs across Africa, the Americas, and Southeast Asia, using recent national Demographic and Health Survey (DHS) data. The findings are intended to support policymakers, healthcare providers, and researchers in developing approaches to strengthen diabetes screening, promote earlier diagnosis, prevent complications, and reduce the burden of undiagnosed diabetes in resource‑limited settings. 

## Methods

### Data source

This study used nationally representative data from the DHS Program, a global health and population research initiative funded by the United States Agency for International Development (USAID) [18]. The DHS Program is conducting cross-sectional household surveys in over 90 LMICs, collecting data on key health indicators. For this analysis, we included the most recent DHS datasets from 14 countries across Africa (Benin 2017-18, Cameroon 2018, Gabon 2019, Gambia 2019, Madagascar 2021, Mali 2023-24, Mauritania 2019, Angola 2023-24, Democratic Republic of Congo 2023, Zambia 2024, and Lesotho 2023-24), North America (Dominican Republic 2013), and Asia (India 2019-2 and Timor-Leste 2016), chosen based on whether diabetes screening uptake data were collected.

### Sampling procedure

As detailed elsewhere [19], the DHS employ a two-stage stratified cluster sampling design. In the first stage, the country is stratified by administrative regions and place of residence (urban/rural), and then clusters (enumeration areas) are selected with probability proportional to their size within each sampling stratum. In the second stage, a complete household listing is conducted in each selected EA. From this listing, a systematic random sample of households is selected for participation in the survey. Within sampled households, eligible respondents are identified and interviewed using standardised DHS questionnaires. Sampling weights were applied to account for the complex survey design, selection bias and non-response, to make estimates representative at the national level for each country [20].

### Study population

This study included individuals aged 15 years and older who responded to the diabetes screening uptake question in the DHS conducted across 14 countries. Participants who were pregnant at the time of the survey were excluded from the analysis to avoid overestimation of diabetes screening uptake, as blood glucose testing is routinely incorporated into antenatal care services (Table 1).

**Table 1:** Study sample distribution, dominant diabetes screening approach and policy attributes by country

Country	Year	Sample (unweighted)	Operational policy/strategy/action plan for diabetes	Evidence-based national diabetes guidelines/protocols/standards	Standard criteria for referral	Diabetes registry	Dominant Screening approach	Primary diagnostic test
Angola	2023-24	20,040	No	Not available	Not available	No	Targeted screening, especially of TB patients and TB suspects	Fasting blood sugar
Benin	2017-18	10,064	Yes	Available and partially implemented	Available and partially implemented	No	Targeted (primarily, project-based)	Fasting blood sugar
Cameroon	2018	18,966	No	don’t know	Not available	No	Targeted and free community-based screening campaigns (NGO initiatives)	Fasting blood sugar
Democratic Republic of Congo	2023	37,405	No data available	No data available	No data available	No data available	Targeted screening with community outreach	Fasting blood sugar
Dominican Republic	2013	19,161	Yes	Not available	Available and partially implemented	Yes	Targeted screening with community outreach	Fasting blood sugar, Oral glucose tolerance test
Gabon	2019	14,038	No data available	No data available	No data available	No data available	Targeted screening with community-based free diabetes screening campaigns (NGO initiatives)	Couldn’t confirm
Gambia	2019	9,919	No	Not available	Not available	No	Targeted and integrated screening (supported by NGO initiatives)	Not generally available
India	2019-21	754,202	Yes	Available and fully implemented	Available and fully implemented	Yes	Population-based screening of all adults aged 30 and opportunistic screening in public health facilities	Fasting blood sugar Oral Glucose Tolerance Test
Lesotho	2023-24	6,422	Yes	Available and partially implemented	Available and partially implemented	No	Targeted screening and community-based screening (rural NGO initiatives)	Fasting blood sugar
Madagascar	2021	17,514	Yes	Available and partially implemented	Available and partially implemented	No	Targeted and community-based screening (NGO initiatives)	Oral glucose tolerance test
Mali	2023-24	26,339	Yes	Available and fully implemented	Available and fully implemented	Yes	Targeted and screening campaigns (NGO initiatives)	Fasting blood sugar, Oral glucose tolerance test
Mauritania	2019	12,742	No	Not available	Not available	No	Passive screening and occasional screening campaign (NGO initiatives)	Not generally available
Timor-Leste	2016	4,080	Yes	Not available	Not available	No	Did not have a clearly documented national diabetes screening program; Opportunistic screening endorsed later (only on plan)	Not generally available
Zambia	2024	25,585	Yes	Available and fully implemented	Available and fully implemented	No	Targeted screening and community awareness programmes	Fasting blood sugar
Total	976,477							

*Dominant testing modalities for Diabetes screening are available in primary care facilities

## Measurements

The DHS uses a standardised questionnaire to gather data on demographic and health variables. The questionnaire is translated into the target population’s language in each country where needed. A detailed description of the DHS questionnaire is published elsewhere [21]. All the variables included in this analysis were measured by the standardised questionnaire.

### Dependent variable

The dependent variable was diabetes screening uptake, measured by the DHS question: “Have you ever had your blood sugar tested by a health professional?” Responses were categorised as “Yes”, “No”, and “Don’t know”. The variable was coded as binary (1 = Yes; 0 = No, or do not know). Self-reported diabetes diagnosis was assessed using a follow-up question: “Have you ever been told by a health professional that you have high blood sugar?” Responses were coded as 1 = Yes, 0 = No, or Do not know.

### Explanatory variables

Explanatory variables were grouped into individual-level and community-level factors. At the individual level, characteristics included age (categorised as < 35 years or ≥ 35 years), sex (male or female), educational attainment (no formal education, primary, secondary, or tertiary), and marital status (married/living with a partner or single/separated/divorced/widowed). Socioeconomic indicators included the household wealth index (poor, middle, or rich) and health insurance coverage (yes/no). Behavioural and health-related factors comprised media exposure (yes, if the participant reported exposure to radio, television, or newspapers at least once per week; no, if less than weekly or never), employment status (yes/no), alcohol consumption (yes/no), tobacco smoking (yes/no), and prior history of blood pressure testing (yes/no).

At the community level, as the DHS does not collect direct community-level attributes, proxy contextual variables were derived by aggregating individual-level responses at the cluster level, clusters being operationalised as communities [22]. Particularly, we created three aggregated community-level measures: community education, community poverty, and community media exposure; in addition, place of residence (urban/rural), as recorded in the DHS dataset, was retained as a community-level variable.

To estimate community poverty, we calculated, for each cluster, the proportion of participants from households in the poor wealth quintile (poor and poorest categories combined) from the DHS wealth index. Community education was defined as the proportion of participants in each cluster who had attained at least secondary education. Community media exposure was computed as the proportion of participants within each cluster who reported exposure to at least one form of mass media (radio, television, or newspapers) at least once per week. Such cluster-level aggregations and their subsequent use in multilevel models have precedent in DHS-based research. After these proportions were generated, each aggregated indicator was dichotomised using the mean when the distribution was approximately normal and the median when it was skewed. Clusters with values at or above the median/mean were classified as having “high,” and those below as “low” for the respective community-level variable. The place of residence remained the binary urban/rural variable as provided.

### Data analysis

After downloading all standard DHS data released as of November 10, 2025, data were processed and analysed using Stata/MP version 19.2 (StataCorp, College Station, TX). Descriptive statistics, such as frequencies and proportions, were conducted to summarise the prevalence of self-reported lifetime diabetes screening uptake by individual and community-level characteristics. We also pooled study-specific weighted prevalence estimates using a random-effects meta-analysis with the empirical Bayes estimator. Pooled prevalence was reported with 95% confidence intervals. Heterogeneity was assessed using Cochran’s Q, τ², and I² statistics. Sensitivity analysis was conducted by excluding participants with known diabetes, and subgroup analysis by region was performed to explore heterogeneity. Inferential statistics, including a multilevel logistic regression, were employed to identify individual- and community-level factors associated with diabetes screening uptake. Multilevel mixed-effects logistic regression models were fitted to account for the hierarchical structure of DHS data, with individuals nested within primary sampling units (PSUs). A two-level structure was specified: individuals at level 1 and communities (PSUs) at level 2. Separate models were run for each country in sequential stages: Null model (Model I): contained no predictors and was used to estimate the extent of between-community variation in diabetes screening uptake. Model II: included only individual-level variables. Model III: included only community-level variables. Model IV (final model): included both individual- and community-level variables. The model was specified as follows:

$$\:logit\left({p}_{\mathcal{c}\mathcal{i}\mathcal{j}}\right)={\beta\:}_{0\mathcal{c}}+\sum_{\mathcal{k}}^{n}{\beta\:}_{\mathcal{k}\mathcal{c}}{X}_{\mathcal{k}\mathcal{c}\mathcal{i}\mathcal{j}}+\sum_{\mathcal{m}}^{\mathcal{n}}{\gamma\:}_{\mathcal{m}\mathcal{c}}{Z}_{\mathcal{m}\mathcal{c}\mathcal{j}}+{u}_{0\mathcal{c}\mathcal{j}}$$

Where, p_cij_ is the probability of diabetes screening uptake for individual i in cluster j in country c, and logit(p_cij_) = ln[p_cij_/(1 − p_cij_)] is the log-odds of the diabetes screening uptake; ϐ_0c_ is the country-specific intercept; χ_kcij_ is the k^th^ individual-level covariate (k = 1,…, K) for individual i in cluster j in country c, with β_kc_ as its corresponding fixed-effect coefficient; Ζ_mcj_ is the m^th^ cluster-level covariate (m = 1,…,M) for cluster j in country c, with ϒ_mc_ as its corresponding fixed-effect coefficient; and υ_0cj_ is the random intercept for cluster j in country c.

Random effects, including community variance, intraclass correlation coefficient (ICC), median odds ratio (MOR) with 95% confidence intervals, and proportional change in variance (PCV), were computed. ICC was computed to determine the proportion of total variation attributable to differences between communities. PCV was calculated across models to assess the reduction in community-level variance resulting from the addition of explanatory variables. Besides, the median odds ratio (MOR) was derived, representing the median increase in the odds of diabetes screening uptake when an individual moves from a lower- to a higher-risk community.

As the Akaike Information Criterion and Bayesian Information Criterion are not directly interpretable for survey-weighted estimation, these indices were calculated from the corresponding unweighted multilevel models and used only to compare alternative model specifications; the survey-weighted models were retained as the primary models for parameter estimation and inference. The variance inflation factor was used to assess multicollinearity among independent variables included in the final model in each survey. A 95% confidence interval and corresponding p-value of less than 0.05 were used to declare statistical significance. Adjusted Odds Ratios (AORs) with corresponding 95% CI were used to estimate the association between diabetes screening uptake and individual and community-level covariates.

## Results

### Sample characteristics

Across the countries included in the analysis, the mean age ranged from 27.69 ± 10.11 years in Angola to 41.13 ± 7.51 years in Timor-Leste, while the mean age among those ever screened ranged from 32.15 ± 10.25 years in Angola to 41.77 ± 7.51 years in Timor-Leste.(see Additional File 1). Women constituted the majority of respondents in most surveys, exceeding 65% in Benin, Cameroon, India, and the Democratic Republic of Congo, while the lowest proportions were observed in Mali (40.6%) and Timor-Leste (43.6%). The proportion of respondents with at least secondary education ranged from 25.5% in Benin to 78.9% in Gabon and 68.0% in the Democratic Republic of Congo. Health insurance coverage was below 10% in most countries, with exceptions in the Dominican Republic (57.0%) and Gabon (67.3%). Media exposure ranged from 34.9% in the Democratic Republic of Congo to 92.7% in the Dominican Republic, with relatively high exposure (≥ 58%) in Gabon, Gambia, and Zambia. The proportion of adults who had ever had their blood pressure measured ranged from 32.4% in Mauritania to 69.5% in the Dominican Republic. The percentage of adults who had ever been told they had high blood pressure varied from 6.0% in Cameroon to 14.7% in Gabon, 14.5% in Timor-Leste, and 14.4% in the Dominican Republic. The proportion of participants with a self-reported diagnosis of elevated blood sugar diagnosed by a health professional was below 5% across all surveyed countries, ranging from 0.6% in Madagascar to 3.8% in the Dominican Republic (see Additional File 1).

At the community level, high community poverty ranged from 11.3% in Gabon to 53.2% in Mali, while high community education ranged from 33.3% in Mali to 80.8% in Madagascar. High community media exposure ranged from 42.4% in Cameroon to 81.6% in Madagascar (see Additional File 1).

### Diabetes screening uptake by country

**Fig. 1:**
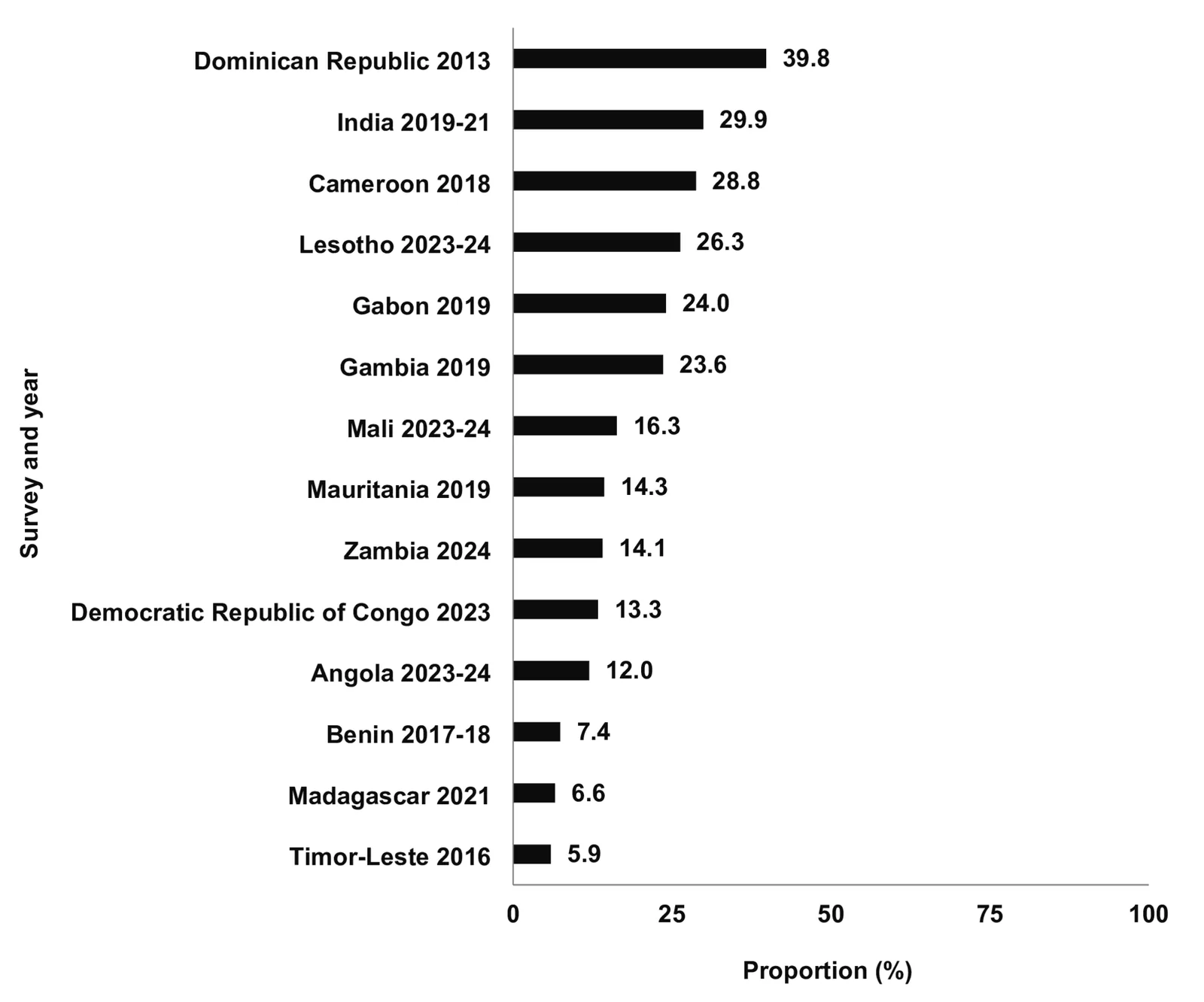
Diabetes screening uptake among adults in middle and low-income countries

The uptake of diabetes screening varied significantly across the countries included in this study, ranging from 5.9% in Timor-Leste to 39.8% in the Dominican Republic, followed by India (29.9%) and Cameroon (28.8%) (Fig. 1). Descriptively, countries with operational plans, implemented national guidelines, referral standards, and diabetes registries generally reported higher screening uptake than those without these supports. Countries relying mainly on targeted, passive, or NGO-supported campaign-based screening approaches tended to show lower uptake, whereas India, which reported population-based screening, had comparatively higher coverage. However, this pattern was not consistent across all countries. Additionally, countries offering both fasting blood sugar and oral glucose tolerance testing at their primary health care level had higher diabetes screening uptake than those relying on fasting blood sugar alone or with limited testing availability (Table 1).

**Fig. 2:**
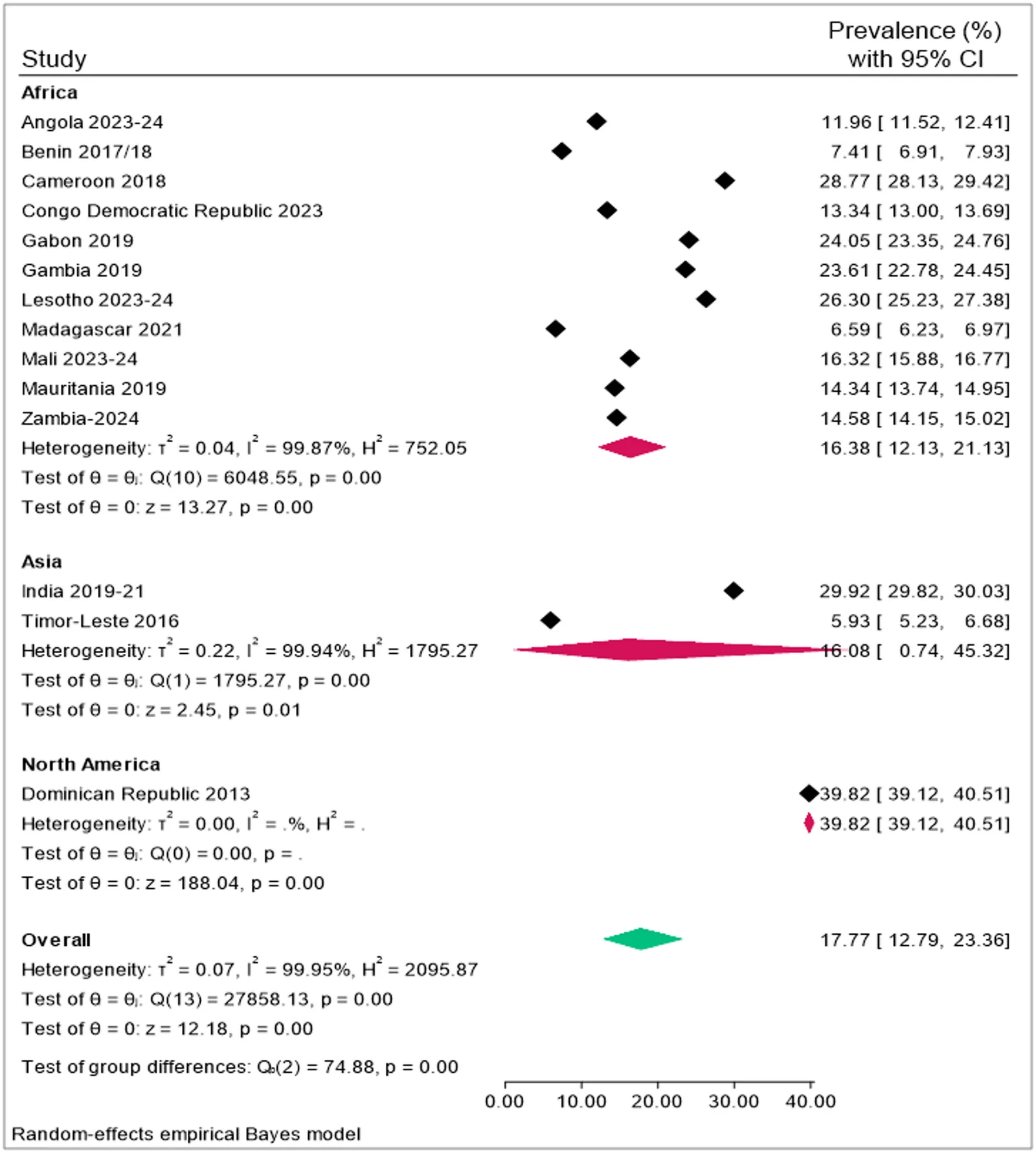
Pooled prevalence of diabetes screening uptake among adults in low and middle-income countries by WHO regions (Note: CI = confidence interval; I² = proportion of total variation across studies attributable to between-study heterogeneity; τ² = between-study variance; H² = ratio of total variability to sampling variability; Q = Cochran’s heterogeneity statistic; Q_b = between-group heterogeneity statistic; p = p-value; z = standard normal test statistic.)

The random-effects meta-analysis showed a pooled prevalence of diabetes screening uptake was 17.8% (95% CI 12.8% to 23.4%), with extreme heterogeneity across studies (I² = 99.95%). In subgroup analysis, pooled prevalence was 16.38% in Africa, 16.08% in Asia, and 39.82% in North America, with significant differences between regions (Q_b_ [2] = 74.88, *p* < 0.001) (Fig. 2). In the sensitivity analysis excluding participants with known diabetes, the pooled prevalence decreased slightly to 16.5% (95% CI 11.7% to 22.0%), while heterogeneity remained extremely high (I² = 99.95%). Corresponding subgroup estimates were 15.3% in Africa, 14.4% in Asia, and 37.4% in North America, and subgroup differences remained significant (Q_b_ [2] = 69.1, *p* < 0.001). These findings indicate that the exclusion of participants with known diabetes did not significantly alter the pooled estimate nor account for the observed heterogeneity (Additional file 5).

### Factors associated with diabetes screening uptake

Across all 14 surveys, the null models (with no covariates) showed substantial community-level variation in diabetes screening uptake, with community variances ranging from 0.28 to 3.97 and corresponding ICC values ranging from 5.3% to 54.7%. Community variance and MOR values declined in most countries after adding individual- and community-level covariates, while PCV increased, reaching 20–50% in several surveys. Model comparison using deviance, AIC, and BIC showed consistent decreases from Model I to Model IV across all surveys, indicating improved model fit with the inclusion of covariates. Hence, Model IV, which included both individual- and community-level covariates across all surveys, was selected as the final model to interpret the results. The full multilevel regression analysis results are presented in Excel (See additional file 3). Moreover, sensitivity analyses were conducted for each survey, showing that the associations for individual-level variables were robust when community-level variables were specified as continuous rather than categorical; however, some community-level associations varied across model specifications (Additional file 4).

As it is presented on the table in the Additional file 2, in the final multilevel logistic regression model, individual-level factors such as older age, female gender, higher educational attainment, being married/living with a partner, higher wealth index, health insurance coverage, and media exposure were significantly associated with increased odds of ever being screened for diabetes across most surveys. High community media exposure, high community education, and urban residence were community-level factors associated with increased odds of ever being screened for diabetes across several surveys (see Additional File 2).

### Individual-level determinants of diabetes screening uptake

Except for Timor-Leste, a one-year increase in the age of the participants consistently demonstrated a higher likelihood of ever being screened for diabetes across all surveys, with adjusted odds ratios (AORs) ranging from 1.03 (95% CI: 1.02–1.04) in Gabon to 1.06 (95% CI: 1.05–1.08) in Lesotho. Male respondents were generally less likely to be ever screened for diabetes compared to females, with the strongest associations observed in Benin (AOR = 0.42; 95% CI: 0.31–0.58), Cameroon (AOR = 0.48; 95% CI: 0.41–0.56), and Mauritania (AOR = 0.59; 95% CI: 0.45–0.78). Educational attainment was a consistent positive predictor of the likelihood of diabetes screening uptake across all surveys. For example, compared with participants with no formal education, those with secondary and tertiary education were more likely to have ever been screened for diabetes in Angola (AOR = 3.58; 95% CI: 2.42–5.30 and AOR = 8.69; 95% CI: 5.52–13.69, respectively) and in Madagascar (AOR = 2.22; 95% CI: 1.48–3.34 and AOR = 5.80; 95% CI: 3.50–9.60, respectively) (see Additional File 2).

Socioeconomic status and marital status were also significantly associated with diabetes screening uptake in most of the surveys. Participants from households in the middle or high wealth quantiles were more likely to be screened for diabetes than those in the poor wealth quantile, with AORs ranging from 1.16 (95% CI: 1.12–1.20) in India to 1.96 (95% CI: 1.38–2.78) in Mauritania. Married or cohabiting adults had higher odds of being screened for diabetes in most surveys, including India (AOR = 2.10; 95% CI: 2.03–2.16), Gabon (AOR = 1.71; 95% CI: 1.41–2.07), and Mauritania (AOR = 1.73; 95% CI: 1.42–2.13). Employed participants had higher odds of being screened for diabetes compared to their counterparts in Angola (AOR = 1.45; 95% CI: 1.20–1.74), Gabon (AOR = 1.34; 95% CI: 1.14–1.58), Gambia (AOR = 1.22; 95% CI: 1.00–1.48), and Lesotho (AOR = 1.31; 95% CI: 1.01–1.69). Health insurance coverage showed a strong positive relationship with diabetes screening uptake across all surveys, with insured participants exhibiting over twice the odds of ever being screened for diabetes in Benin (AOR = 2.13; 95% CI: 1.37–3.30), Cameroon (AOR = 2.29; 95% CI: 1.70–3.09), and Mauritania (AOR = 1.87; 95% CI: 1.44–2.44). Regular exposure to mass media was another factor associated with increased odds of being screened for diabetes in Benin (AOR = 1.36; 95% CI: 1.09–1.70), Madagascar (AOR = 1.58; 95% CI: 1.27–1.98), Mali (AOR = 1.33; 95% CI: 1.15–1.53), Lesotho (AOR = 1.22; 95% CI: 1.00–1.49), Dominican Republic (AOR = 1.30; 95% CI: 1.09–1.55) and India (AOR = 1.05; 95% CI: 1.02–1.07). Moreover, the predicted probabilities showed that diabetes screening uptake was consistently higher among respondents who had ever been tested for blood pressure than among those who had never been tested in all countries included in this analysis, ranging from 6.04% (95% CI: 5.21–6.88) to 48.62% (95% CI: 47.54–49.70) versus 0.95% (95% CI: 0.52–1.39) to 12.00% (95% CI: 10.55–13.45), respectively. (see Additional File 2).

### Community-level determinants

High community poverty was inversely associated with diabetes screening uptake in several countries, including the Gambia (AOR = 0.52; 95% CI: 0.33–0.82), Angola (AOR = 0.52; 95% CI: 0.30–0.91), India (AOR = 0.57; 95% CI: 0.54–0.60), Madagascar (AOR = 0.61; 0.40–0.93), and Mauritania (AOR = 0.70; 95% CI: 0.55–0.90). Conversely, in Benin, individuals residing in high-poverty communities had significantly higher odds of being screened for diabetes (AOR = 1.64; 95% CI: 1.14–2.37). Participants from communities with high community education had increased odds of being screened for diabetes in India (AOR = 1.44; 95% CI: 1.38–1.51) and the Democratic Republic of Congo (AOR = 1.25; 95% CI: 1.03–1.53). Participants from communities with high community media exposure were more likely to be screened for diabetes in Benin (AOR = 2.01; 95% CI: 1.48–2.73), Angola (AOR = 1.51; 95% CI: 1.04–2.72), Cameroon (AOR = 1.51; 95% CI: 1.18–1.94), and India (AOR = 1.88; 95% CI: 1.79–1.97). Participants from rural communities had lower odds of being screened for diabetes in Madagascar (AOR = 0.53; 95% CI: 0.38–0.72) and Mali (AOR = 0.67; 95% CI: 0.52–0.87). On the other hand, rural residence was associated with increased odds of ever being screened for diabetes in India (AOR = 1.07; 95% CI: 1.02–1.13) (see Additional File 2).

## Discussion

Diabetes screening uptake was low among adults across the included LMICs, with lifetime screening prevalence below 25% in most countries. Diabetes screening uptake was associated with a combination of individual-level factors, including age, educational attainment, health insurance coverage, and exposure to mass media, as well as broader community-level factors.

The prevalence of diabetes screening uptake varied widely, ranging from 5.9% in Timor-Leste (2016) and 6.6% in Madagascar (2021) to over 39% in the Dominican Republic (2013). The variation in diabetes screening uptake across countries may partly reflect differences in how screening is organised and implemented. In this study, countries with broader screening models, including population-based, integrated, outreach, or community-based approaches, generally showed higher uptake, whereas countries relying on passive, project-based, high-risk-only, or poorly documented approaches tended to have lower uptake. By contrast, policy availability alone did not consistently correspond to higher screening uptake, suggesting that implementation and programme reach may be more important than the mere existence of policy documents.

The pooled prevalence from the meta-analysis was 17.8% (95% CI 12.8% to 23.4%), indicating that fewer than one in five adults across these settings had ever undergone diabetes screening. These findings align with previous evidence showing that diabetes screening uptake in LMICs remains markedly below global targets and far behind rates reported in high-income countries, where population-based and routine screening is more accessible [15–24]. Consistent with literature from Sub-Saharan Africa and South Asia, women were more likely than men to undergo screening in nearly all countries, a pattern likely attributable to the higher frequency of contact women have with health systems, particularly through maternal and reproductive health services [15, 16]. Indeed, the findings provide important multi-country evidence on diabetes screening uptake across 14 LMICs; however, they should be interpreted with caution, given the limited country coverage. Differences in health systems, diabetes screening policies, population risk profiles, and access to care across LMICs mean that the observed patterns may not be generalisable to all low- and middle-income settings.

The multilevel analysis identified several individual- and community-level factors associated with diabetes screening uptake, including both non-modifiable and potentially modifiable characteristics. Non-modifiable factors, such as age and sex, may help identify population groups with lower screening uptake and inform more targeted screening strategies. At the same time, potentially modifiable or intervention-relevant factors, including health insurance coverage, blood pressure measurement, mass media exposure, and community-level education, may provide opportunities to improve screening coverage.

Increasing age was consistently associated with higher odds of ever being screened for diabetes in all countries included in our analysis (AORs = 1.01–10.7), as expected given the rising risk of metabolic disease with age and the increased likelihood of interacting with health-care providers [25, 26]. Education demonstrated a clear dose-response effect, with individuals with secondary (AORs = 1.29–2.10) or higher (AORs = 1.73–8.69) education exhibiting significantly greater odds of screening uptake than those with no formal schooling. This aligns with evidence showing that health literacy strongly predicts preventive health uptake and risk awareness [26–28]. Similarly, we found that health insurance coverage was significantly associated with the increased likelihood of being screened for diabetes across multiple countries, (AORs = 1.36–3.26), in line with previous studies [29–31]. This may be because insurance reduces out-of-pocket costs and other financial barriers to clinic attendance and laboratory testing, increasing routine contact with primary care where opportunistic screening can occur. This was supported by the findings of a systematic review of 68 studies in LMICs that found that public health insurance coverage generally increases healthcare utilisation and often improves financial protection [32]. These findings also support the universal health coverage (UHC) agenda, which emphasises access to health services, including prevention, without financial hardship [33]. Wealth also positively influenced screening uptake across several countries, reflecting socioeconomic gradients in service access and health-seeking behaviour [27]. Indeed, the positive associations of wealth and health insurance coverage with diabetes screening uptake may reflect the selective or targeted nature of screening delivery in many settings, whereby individuals with greater socioeconomic advantage may be better positioned to access testing.

In our analysis, prior blood pressure measurement was strongly associated with the likelihood of being screened for diabetes across surveys (AORs = 6.33–29.59), consistent with existing evidence of a very large association between blood pressure and blood glucose testing [30, 31]. This association may be due to increased contact with healthcare services: individuals who have their blood pressure checked may be more likely to receive health education and counselling about chronic disease risk, which can raise health awareness and perceived susceptibility to conditions such as diabetes. This increased perceived risk may, in turn, prompt uptake of diabetes screening [34]. This association may also reflect the fact that hypertension and diabetes are co-components of metabolic syndrome, share upstream causes like central obesity/insulin resistance, and are routinely bundled in clinical screening and follow-up guidelines, so the same high-risk individuals tend to receive both tests [35].

In our analysis, the community-level poverty, education, and media exposure were significantly associated with the likelihood of diabetes screening uptake. Consistent with previous studies, we found that high community poverty was associated with lower odds of diabetes screening uptake in several countries, including Angola, The Gambia, India, Madagascar, and Mauritania, with AORs ranging from 0.52 to 0.70 [36]. However, in Benin (AOR = 1.64), it was associated with increased screening uptake. This may be because, in some settings, higher community poverty may coincide with higher screening uptake when screening is delivered through pro-poor community platforms (community health workers, community relays, outreach campaigns) that predominantly operate in underserved areas [37, 38].

Media exposure, both at the individual and community levels, was also strongly associated with screening uptake across several countries in this analysis. Individuals exposed to mass media (radio, television or newspaper) at least weekly had significantly higher odds of being screened for diabetes in India, Benin, Madagascar, Lesotho, Angola, and Mali (AORs = 1.05–1.58). Besides, Adults from communities with high mass media exposure had higher odds of being screened for diabetes in Benin (AOR = 2.01), Cameroon (AOR = 1.51), India (AOR = 1.88), Angola (AOR = 1.68) and Zambia(AOR = 1.40), suggesting that community information environments shape collective health behaviours. These findings align with the literature demonstrating that mass-media campaigns, health messaging, and social diffusion of health knowledge contribute meaningfully to Non-Communicable Diseases (NCDs) prevention [39, 40]. This highlights that the use of mass media for health communication could be a less expensive but effective strategy to combat undiagnosed/ late diagnosed diabetes and its health and socioeconomic consequences.

High community education was associated with higher odds of diabetes screening uptake across several settings, including India and the Democratic Republic of Congo (AOR = 1.25–2.58). This may be because higher community education may reflect greater access to health services (often correlated with urbanisation) and a more supportive information environment, such as stronger prevention norms and more effective diffusion of health information, which could facilitate diabetes screening uptake [41–43].

Random-effects components in the multilevel models indicated substantial between-community variation, with intraclass correlation coefficients ranging from 8% to over 40% across countries. Countries such as India and Madagascar exhibited particularly high community-level clustering, indicating shared contextual factors such as community-level health communication environments, and socioeconomic factors strongly shape diabetes screening uptake [36, 44].

### Public health implications

First, the low prevalence of lifetime diabetes screening uptake observed across the countries included in this analysis indicates substantial missed opportunities for the early detection of hyperglycaemia, which is predominantly asymptomatic in its early stages [4]. This is particularly concerning given that approximately 40% of the adult population in LMICs is estimated to be at increased risk of diabetes, while only 60%-63% of individuals with diabetes in LMICs report ever having had their blood glucose tested [11, 12, 45]. Enhancing health systems’ diabetes screening capabilities and increasing community awareness of chronic conditions could help prevent avoidable complications, avoidable health expenditures, and mortality associated with undiagnosed or late-stage diabetes.

Second, the association between hypertension and diabetes screening uptake suggests that integrated NCD service delivery may offer an important opportunity to expand diabetes screening coverage. While the cross-sectional nature of the data precludes conclusions about temporal sequence, routine blood pressure measurement contacts at primary care and community levels may provide feasible platforms for strengthening diabetes screening efforts [35]. Third, the socioeconomic and educational disparities observed indicate the need for multilevel interventions that address both structural and informational barriers to diabetes screening. Policies aimed at improving financial protection, promoting health literacy, and advancing gender-responsive service delivery may be important for reducing inequities in screening uptake [32]. Finally, the significant community-level variability in diabetes screening uptake and the significant community-level clustering suggest that community-specific interventions, including media-based health promotion, community health worker outreach, and strengthening local health systems, may be effective in improving diabetes screening uptake in LMICs [38, 46].

### Strengths and limitations of the study

This study has several notable strengths. Foremost, it draws on recent, nationally representative DHS data from multiple LMICs, allowing for a comprehensive assessment of diabetes screening uptake across diverse epidemiological, social, and health system contexts. The large sample size provides substantial statistical power to identify both individual- and community-level factors associated with screening practices. Besides, the application of survey weights could enhance the representativeness of the findings to each target population by adjusting for selection and non-response biases. Moreover, hierarchical multilevel modelling enhances analytic rigour by appropriately accounting for the nested structure of DHS data and quantifying between-community variability in diabetes screening uptake. This approach permits a more accurate estimation of the influence of contextual factors, such as community poverty, education, and media exposure, on screening uptake.

Despite these strengths, the findings of this manuscript should be interpreted with several limitations in mind. First, diabetes screening uptake was self-reported, which may introduce recall or social desirability bias and potentially misclassify individuals’ true screening uptake [47]. In addition to recall bias, participants might have undergone blood glucose testing without realising it was for diabetes screening, which could have influenced our estimates of the prevalence of diabetes screening. Second, because the DHS relies on cross-sectional designs, the causal associations cannot be determined. Third, although the analysis included important individual and community-level covariates, unmeasured confounders, such as health system factors (e.g., availability of diagnostic services, access to healthcare services), cultural perceptions of chronic disease, and individual knowledge and risk perception of diabetes, may still influence the observed relationships. Fourth, while the multilevel models account for community-level clustering, the proxies for contextual characteristics (e.g., community poverty or media exposure) may not fully capture the complex social and environmental determinants of diabetes screening uptake. Fifth, the outcome, ever having been screened for diabetes, was based on self-reported lifetime experience without a clearly defined reference period, which may limit cross-country comparability given differences in the timing of DHS data collection across settings. Sixth, DHS are primarily designed to represent populations of reproductive age; the analytic sample was skewed toward younger adults, with limited representation of older individuals. Consequently, the prevalence of self‑reported diabetes observed in this analysis was substantially lower than country‑specific estimates derived from other data sources, reflecting the well‑documented increase in diabetes prevalence with advancing age [2]. Nevertheless, the age composition of the sample does not negate the relevance of the findings for understanding diabetes screening uptake in these settings, as type 2 diabetes increasingly affects younger adults and delayed diagnosis in this population remains a public health concern. In addition, emerging evidence has described a malnutrition‑related form of diabetes (“type 5 diabetes”) that disproportionately affects younger individuals, often under 30 years of age, and is thought to be more prevalent in LMICs. This further underscores the importance of assessing diabetes screening uptake among younger adults in this context to design relevant diabetes screening strategies through primary care systems and/or community outreach programmes. Lastly, although each DHS survey is nationally representative within its respective country, the findings should not be interpreted as representative of all LMICs. Generalisability is limited because country inclusion was determined by the availability of DHS diabetes screening data, and the included countries may not capture the full geographic, socioeconomic, health-system, and epidemiological diversity of LMICs. Therefore, the findings are most applicable to the 14 included countries and to similar LMIC settings.

## Conclusion

Diabetes screening uptake among adults in LMICs was very low and significantly variable across countries. Individual-level factors such as age, gender, media exposure, education attainment, and family wealth, as well as blood pressure checks, were important factors affecting diabetes screening uptake, along with community-level factors such as community education, community media exposure, and community-level poverty in most contexts. There was significant variability in diabetes screening uptake across communities in all surveys included, highlighting the importance of contextual factors in promoting early detection of diabetes. The study underscores that increasing routine opportunistic screening in primary care through hypertension screening platforms, improving access to health services, and disseminating health information through mass media could enhance diabetes screening uptake, promote earlier detection, and reduce the burden of undiagnosed diabetes in these resource-limited settings.

## Supplementary Information

Additional File 1. Individual and community-level characteristics of respondents by diabetes screening uptake.

Additional file 2. Mixed-effects multilevel logistic regression analysis of factors affecting diabetes screening uptake.

Additional file 3. Mixed effects analysis of factors affecting diabetes screening uptake (full analysis).

Additional file 4 (xlsx): Sensitivity analysis of the multilevel regression model using community-level variables specified as continuous measures.

Additional file 5 (docx): Sensitivity analysis for the meta-analysis of pooled diabetes screening uptake prevalence across 14 low- and middle-income countries.

## Data Availability

All datasets used in this study are publicly available through the DHS Program website ( https://dhsprogram.com ) upon registration and approval.

## Abbreviations

AIC: Akaike Information Criterion
AOR: Adjusted Odds Ratio
BIC: Bayesian Information Criterion
CI: Confidence Interval
DHS: Demographic and Health Survey
HbA1c: Glycated haemoglobin
ICC: Intra-Class Correlation
LMICs: Low- and Middle-Income Countries
MOR: Median Odds Ratio
PCV: Proportional Change in Variance
T2D: Type 2 Diabetes
UHC: Universal Health Coverage
WHO: World Health Organisation
