# Supplementary material for: Individual and community-level factors affecting diabetes screening uptake among adults in 14 low- and middle-income countries: a multilevel mixed-effects analysis of recent demographic and health surveys
Source: BMC Public Health. 2026 Jul 10;26:2784. doi: 10.1186/s12889-026-28221-3 (PMC13637119; doi:10.1186/s12889-026-28221-3)
Supplement: Supplementary file 1 [file 12889_2026_28221_MOESM5_ESM.docx]

**Additional file 5 (docx): Sensitivity analysis for the meta-analysis of pooled diabetes screening uptake prevalence across 14 low- and middle-income countries**
